# Current status of clinically available bioresorbable scaffolds in percutaneous coronary interventions

**DOI:** 10.1007/s12471-015-0652-2

**Published:** 2015-01-28

**Authors:** Cordula Felix, Bert Everaert, Roberto Diletti, Nicolas Van Mieghem, Joost Daemen, Marco Valgimigli, Peter P. de Jaegere, Felix Zijlstra, Evelyn Regar, Cihan Simsek, Yoshinobu Onuma, Robert-Jan M. van Geuns

**Affiliations:** Department of Cardiology, Thoraxcentre, Room Ba-585, Erasmus University Medical Centre, ‘S-Gravendijkwal 230, 3015 Rotterdam, GE The Netherlands

**Keywords:** Review, Bioresorbable, ABSORB BVS, PCI, Coronary artery disease

## Abstract

Drug-eluting stents (DES) are widely used as first choice devices in percutaneous coronary interventions. However, certain concerns are associated with the use of DES, i.e. delayed arterial healing with a subsequent risk of neo-atherosclerosis, late stent thrombosis and hypersensitivity reactions to the DES polymer. Bioresorbable vascular scaffolds are the next step in percutaneous coronary interventions introducing the concept of supporting the natural healing process following initial intervention without leaving any foreign body materials resulting in late adverse events. The first-generation devices have shown encouraging results in multiple studies of selected patients up to the point of full bioresorption, supporting the introduction in regular patient care. During its introduction in daily clinical practice outside the previously selected patient groups, a careful approach should be followed in which outcome is continuously monitored.

## Introduction

Drug-eluting stents (DES) are widely used as devices of first choice in percutaneous coronary intervention (PCI). However, certain concerns are associated with the use of DES, i.e. delayed arterial healing with a subsequent risk of neo-atherosclerosis, late stent thrombosis and hypersensitivity reactions to the DES polymer [1].

Furthermore, from a more general physiological point of view, a vessel that is indefinitely caged in a metal scaffold is not desirable in both the short and the long term, because of the risk of impaired endothelial function, the reduced potential for vessel remodelling, interference with the normal arterial healing process and the risk of occlusion of covered side branches by neointima hyperplasia. Also, interference with non-invasive imaging (cardiac computed tomography or magnetic resonance imaging) during patient follow-up and possible impairment of future treatment options (re-PCI or coronary artery bypass surgery) are drawbacks of metallic stents [2]. Therefore, a stent type made of a bioresorbable material could provide the desirable transient vessel support without compromising the restoration of normal vessel biology, vessel imaging or treatment options in the long run. Furthermore, the need for long-term dual antiplatelet therapy could potentially be reduced.

The Igaki-Tamai stent was the first-in-man fully biodegradable coronary stent made of poly-L-lactic acid (PLLA). However, this stent did not possess any active antiproliferative drug coating and this resulted in an unacceptably high early target vessel revascularisation rate. On the other hand, late invasive follow-up confirmed the fully bioresorption process and coverage of complex atherosclerotic lesions with a stable layer of neo-media. From September 1998 until April 2000, 50 patients were treated. Data of 10-year follow-up showed high first-year target vessel failure with acceptable rates of major adverse cardiac events during the late follow-up [3].

The Absorb Bioresorbable Vascular Scaffold (BVS, Abbott Vascular, Santa Clara, CA) consists of a PLLA bioresorbable scaffold with poly D, L-lactide bioresorbable (PDLLA) coating that releases the antiproliferative drug everolimus. The long chains of PLLA and PDLLA are degraded via hydrolysis of the ester bonds and the resulting lactate and its oligomers are metabolised by the pyruvate and Krebs energy cycles. Two adjacent radio-opaque platinum markers are located at both Absorb edges to allow long-term visualisation. The strut thickness is approximately 150 µm.

It has been suggested that patients treated with a BVS need more aggressive antiplatelet therapy because of these thicker struts. Prasugrel, a third-generation thienopyridine prodrug, induces platelet inhibition more consistently and to a greater extent than clopidogrel which resulted in less stent thrombosis, urgent target vessel revascularisation and myocardial infarction at the cost of a small increase in major bleeding in the randomised controlled PLATO study [4]. The Rijnmond Collective Cardiology Research registry is a prospective, observational study that will assess the adaption of prasugrel into routine clinical practice and in the near future will deliver real-world numbers about reducing ischaemic events on one hand and the increased risk of bleeding on the other hand [5]. If safety is confirmed in this routine clinical practice, prasugrel might be the preferred treatment for patients treated with BVS.

The Absorb Bioresorbable Vascular Scaffold was the first fully bioresorbable scaffold to receive a CE mark. A comparable PLLA-based scaffold coated with myolimus has completed its first-in-man study with encouraging results and also obtained a CE mark [6]. With its current limited scientific evidence of efficacy, this review will concentrate on the only widely available BVS, the Absorb scaffold.

In a parallel publication in this journal recommendations for the general usage of the BVS are presented [7].

## The beginning of BVS: ABSORB Cohort A and B

The ABSORB Cohort A was the first-in-man trial to investigate the safety and feasibility of the everolimus-eluting BVS. In this prospective, multicentre, single-arm, open-label trial 30 patients with stable, unstable or silent ischaemia were enrolled from March until July 2006. Coronary lesions had to be single and *de novo* in a native coronary artery with a stenosis of > 50 % and with a TIMI flow grade > 1.

Major exclusion criteria were ST-elevation myocardial infarction (STEMI) patients, patients presenting with unstable arrhythmias or those with a ventricular ejection fraction < 30 %. Significant stenosis in the left main coronary artery, lesions involving a side branch > 2 mm in diameter and lesions with the presence of thrombus or more than one clinically significant stenosis in the target vessel were excluded.

The clinical endpoints were assessed at 30 days, 6 and 9 months and 1, 2, 3, 4 and 5 years and were excellent. Except from one non-Q-wave myocardial infarction, no other major adverse cardiac events were noted in up to 2 years (defined as cardiac death, myocardial infarction and ischaemia-driven target lesion revascularisation).

**Fig. 1:**
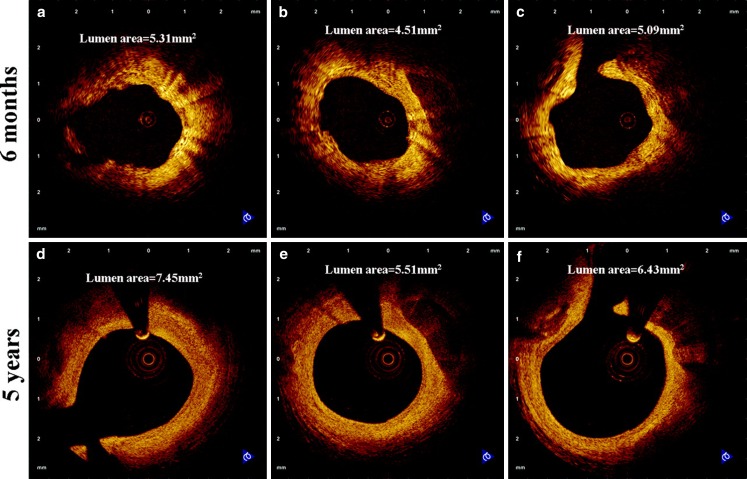
Optical coherence tomography images of coronary arteries from matched sites at 6 months (**a**–**c**) and 5 years (**d**–**f**) after BVS implantation

After 2 years, invasive coronary imaging studies showed that the BVS were largely absorbed and had been incorporated into the vessel wall. The remaining strut parts were apposed and late lumen enlargement could be demonstrated. Vasomotion and endothelial function were evaluated after intracoronary injection of methergin (a vasoconstrictor) and acetylcholine (an endothelium-dependent vasodilatator). This confirmed restoration of normal endothelium-dependent vessel wall function after degradation of the vascular scaffold ([8], Fig. 1).

A 5-year clinical follow-up was obtained in 27 patients (one patient withdrew consent and two patients died of a non-cardiac cause). The major adverse cardiac event rate at 5-year follow-up was low (3.4 %). No scaffold thrombosis was reported [9]. In ABSORB Cohort A the first-generation device was used. Tanimoto et al. described that acute stent recoil was slightly but insignificantly larger when compared with that of the everolimus-eluting stent (6.9 vs 4.3 %) [10]. This first-generation BVS showed a late lumen loss of 0.44 mm, probably due to device shrinkage. To overcome the potential issue of acute scaffold recoil, a second-generation BVS with a modified scaffold design was tested in the ABSORB Cohort B trial. This revised scaffold was developed to provide greater vessel wall support, a more consistent drug delivery and device storage at room temperature.

The ABSORB Cohort B trial had a prospective, multicentre, single-arm, open-label design. In total 101 patients were included and subdivided into two groups according to the invasive imaging protocol. The first group (B1, *n* = 56) underwent angiography at 6 and 24 months and the second group (B2, *n* = 45) received follow-up angiography at 12 and 36 months. Also, during angiography, the implanted scaffolds were additionally investigated with intravascular ultrasound and optical coherence tomography. In ABSORB Cohort B, patients with a maximum of 2 *de novo* coronary artery lesions were included (maximum lesion diameter and length of 3.0 and 14 mm, respectively, for a scaffold size of 3.0 × 18 mm). The other inclusion and exclusion criteria did not differ from the ABSORB Cohort A trial. At 3-year follow-up there were no cases of cardiac death or scaffold thrombosis, three cases of myocardial infarction (all non-Q-wave), and seven ischaemia-driven target lesion revascularisations with a major cardiac adverse event rate of 10 %. No scaffold thrombosis was evident during follow-up [11].

**Fig. 2:**
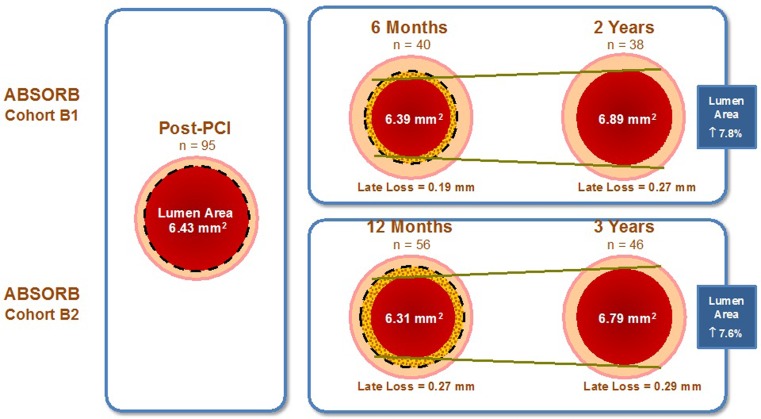
Evolution of the IVUS-measured mean lumen area in coronary arteries treated with BVS in ABSORB Cohort B1 and B2

Imaging with intravascular ultrasound demonstrated late lumen enlargement of the scaffolded lesions in the ABSORB Cohort A and B patients. This observation could represent a paradigm shift from late lumen loss to late lumen gain when applying BVS technology ([12], Fig. 2).

Also, results on restoration of vasomotor function were reported for the ABSORB A and B Cohorts. These data suggest a progressive recovery of normal vascular function in the scaffolded segments during the resorption process [13].

Recently Karanos et al. reported about the long-term vascular healing response of eight patients from the ABSORB Cohort A. Five years after BVS implantation patients underwent invasive follow-up with optical coherence tomography, revealing late luminal enlargement, complete strut bioresorption and development of a ‘sealing layer’ covering underlying thrombogenic plaque components [14].

In brief, the ABSORB Cohort A and Cohort B trial included only non-complex lesions with low-risk patients. Placement of BVS proved to be feasible and safe, with major adverse cardiac events and stent thrombosis rate similar to Xience V. Based on the clinical safety demonstrated in the first studies (ABSORB Cohort A and B), the everolimus-eluting BVS acquired a CE mark in Europe and has since become commercially available. However, to further expand the indication for BVS use in more complex coronary lesions and acute coronary syndrome patients, the BVS Expand, ABSORB Extend and ABSORB II and BVS STEMI first study, respectively, were initiated.

## Extend clinical evaluation of BVS

To explore the performance of BVS in a larger group of patients with different operators, the ABSORB Extend study was initiated in more than 100 non-US sites worldwide. This continued access, non-randomised, prospective, single-arm clinical trial was started in January 2011 and intended to include more than 800 patients with up to 2 *de novo* lesions in different epicardial vessels. The range of scaffold diameters and sizes was extended (2.5, 3.0 and 3.5 mm in diameter and 12, 18 and 28 mm in scaffold length, respectively) to allow the treatment of a broader range of coronary lesions (≤ 28 mm in length and reference vessel diameter of 2.0–3.8 mm (as assessed by on-line quantitative coronary angiography or intravascular ultrasound)). One stent overlap was allowed for lesions of more than 22 and less than 28 mm. Target lesions located in the left main coronary artery, arterial or saphenous vein grafts, in-stent restenosis, lesions previously treated with brachytherapy, chronic total occlusions (TIMI 0 prior to wire crossing), bifurcation lesions with side branches ≥ 2 mm in diameter, ostial lesion of 40 % stenosis or a side branch requiring predilatation were excluded from the study. Also lesions with excessive calcification, high tortuosity or visible thrombus were excluded.

Recently, an interim analysis on the 12-month clinical outcome of the 512 first BVS implanted patients demonstrated a favourable clinical outcome and safety profile (Table 1). Cardiovascular death, ischaemia-driven major adverse cardiac events and target vessel failure occurred in 0.4, 4.3 and 4.9 % of patients, respectively. The incidence of scaffold thrombosis was low (0.8 %) [15]. Propensity-matched clinical outcomes at 1 year showed identical cardiovascular death, hierarchical major adverse cardiac event and stent thrombosis rates for BVS compared with second-generation DES (Xience V) (0.3 vs. 0.6 %, 5.2 vs. 5.5 % and 0.5 vs 0.5 %, respectively) [16]. Interestingly, in a propensity score analysis comparison between Absorb Cohort B/Extend patients and Xience V patients from Spirit Cohorts, target vessel failure rates were significantly lower in BVS compared with DES (5.5 vs. 8.6 %, respectively, *p* = 0.04). A 2-year follow-up propensity-matched analysis confirmed the non-inferiority of BVS compared with Xience V [17].

**Table 1:** Overview of currently reported BVS studies and registries

	EXTEND	ASSURE	ABSORB FIRST	EXPAND	AMC	Milan	GHOST-EU	BVS STEMI first	Mainz ACS	Polar ACS	Prague-19
N	512	183	800	200	135	92	1189	49	150	100	76
Sites	56	6	95	1	1	2	10	1	1	Multi	2
Period	1/10–12/12	4/12–3/13	1/13–3/14	9/12–10/13	8/12–8/13	5/12–8/13	11/11–1/14	11/12–4/13	5/12–6/13	11/12-10/13	12/12–4/14
ACS	0 %	21.3 %	38 %	60.4 %	50 %	10.9 %	47.4 %	100 % all STEMI STESTEMI)	100 %	100 %	100 % all STEMI STESTEMI_STEMI)
Single vessel PCI	93 %	–	90.7 %	61.5 %	81.1 %	–	–	100 %	–	100 %	–
Lesions/patient	1.1	1.1	1.2	1.4	1.2	1.5	1.2	1.0	1.2	1.0	1.2
Lesion length	11.9 mm	15 mm	18.3 mm	25.4 mm	–	36.5 mm	19.4 mm	26.4 mm	19.4 mm	–	23.2 mm
Calcification (moderate/severe)	15 %	15.7 %	20.4 %	45.8 %	11.3 %	20.4 %	–	–	–	–	–
B2	41 %	43.4 %	23.1 %	24.4 %	42.1 %	83.9 % (B2 + C)	23.6 %	–	–	–	–
C	2 %	21.2 %	23.6 %	16.7 %	25.2 %		27.6 %	–	–	–	–
Device success	98.6 %	–	98.9 %	98.2 %	96 %	–	99.7 %	97.9 %	–	100 %	96.2 %
TLR	1.8 % at 1 year	2.8 % at 1 year	–	2.2 % at 6 months	5.0 % at 6 months	3.3 % at 6 months	2.5 % at 6 months	0 % at 30 days	2.0 % at 30 days	0 % at 1 year	1.3 % average of 6 months
TVR	–	–	–	2.2 % at 6 months	6.6 % at 6 months	3.3 % at 6 months	4.0 % at 6 months	0 % at 30 days	–	1.1 % at 1 year	–
Definite scaffold thrombosis	0.8 % at 1 year	0 % at 1 year	0.3 % at 30 days	2.2 % at 6 months	3.2 % at 6 months	0 % at 6 months	1.7 % at 6 months	0 % at 30 days	2.0 % at 30 days	1.1 % at 1 year	1.3 % average of 6 months
MACE	4.3 % at 1 year	5 % at 1 year	–	3.3 % at 6 months	–	3.3 % at 6 months	–	2.6 % at 30 days	6.6 % at 30 days	3 % in-hospital	2.6 % average of 6 months

Interestingly, the results from a propensity-matched analysis of 250 patients, comparing patients implanted with BVS with patients implanted with Xience V in the SPIRIT IV trial, showed a decrease in angina pectoris reported by the sites through adverse event reporting at 1 year (16.0 vs 28.1 %, respectively) [18]. This difference was highly significant and probably accounts for the lower target vessel failure rate in the BVS group. Also, the percentage of angina diagnosed through adverse event reporting was notably lower with BVS than that reported in previous large interventional trials (FREEDOM (sirolimus-eluting stent/paclitaxel-eluting stent): 21 %; SYNTAX (paclitaxel-eluting stent): 28 %; COURAGE (bare metal stent: 34 %) [16]. Further follow-up is needed to confirm this observation on the potential reduction of post-PCI angina. If confirmed, repeat angiography with or without additional coronary intervention would be significantly reduced. This could greatly impact on patient quality of life and additionally reduce healthcare costs.

The ABSORB II study started in November 2011 as the first randomised (2:1), prospective, single-blinded, multicentre trial, in which patients were assigned to the ABSORB BVS or a second-generation everolimus-eluting coronary stent (Xience Prime). A total of 501 patients were randomised across 40 European sites and in New Zealand. Patients with stable or unstable angina, silent ischaemia and with up to 2 *de novo* lesions in different epicardial vessels with a maximal lesion length of 48 mm were enrolled. Major exclusion criteria were STEMI, left ventricular ejection fraction < 30 %, unstable arrhythmias, left main disease, chronic total occlusions and severely calcified or tortuous lesions. Patients will be followed for 5 years, with an invasive evaluation by angiography, intravascular ultrasound, optical coherence tomography, and vasomotion testing at final follow-up for superiority [19]. First one-year interim analysis showed non-inferiority between BVS and DES on major adverse cardiac events which is essential to achieve the superiority endpoint [20].

## BVS in more complex coronary lesions in everyday patients

In September 2012, at the Erasmus MC, the Expand registry was initiated to evaluate the long-term safety and performance of the BVS in routine clinical practice. In this monocentre, prospective, observational registry, patients presenting with non-ST-elevation myocardial infarction (NSTEMI), stable or unstable angina or silent ischaemia in combination with a *de novo* stenotic lesion in a native, previously untreated, coronary artery were included. A reference vessel diameter up to 4 mm and a longer lesion length (> 32 mm) was allowed, as was a higher degree of calcification and bifurcation lesions. Major exclusion criteria were previous coronary artery bypass graft or metallic stent in the target vessel, cardiogenic shock, STEMI, bifurcation lesions requiring kissing balloon post-dilatation, allergy or contraindications to dual antiplatelet therapy. In the first 200 patients, on average 1.9 scaffolds were implanted per patient, with stent overlap in 32 % of patients. The mean lesion length was 25.4 ± 13.5 mm. Of the lesions 41.1 % were scored as B2 or C lesions, 5.8 % were chronic total occlusions and in 29.1 % a bifurcation was included. Of the patients 38.5 % had multi-vessel disease. The procedural success rate of BVS implantation was 98.2 %, with a radial approach in 76.6 % and lesion preparation in 91.9 % of lesions (275 in total). The 6-month results were excellent with a mortality of 2 %, a definite scaffold thrombosis of 2.2 % and no other target lesion revascularisation within this period. Final rate of major adverse cardiac events at 6 months was 3.3 % (Table 1, [21]).

Recently, the 6-month outcome data of the Italian all-comer patient GHOST-EU registry, including 1189 patients with moderate to high complex lesion and/or patient characteristics, were reported, showing acceptable rates of cardiovascular death (1.0 %), target vessel myocardial infarction (2.0 %) and of target lesion failure (4.4 %) [22]. Definite scaffold thrombosis rates were 1.7 % at 6 months. Also, the Academic Medical Centre single-arm first experience, including a high number of complex patients, showed a somewhat higher major adverse cardiac event rate at this time point, especially related to scaffold thrombosis. The investigators claim that this was due to a learning curve where major changes were made with regard to lesion preparation and post-dilatation to achieve full scaffold expansion and avoiding underexpansion as observed in the first scaffold thrombosis cases [23]. Conversely, a propensity-matched analysis from the single centre San Raffaele Scientific Institute BVS registry (Milan, Italy), comparing BVS (*n* = 92) with Xience V (*n* = 92) in complex lesions (83.9 % B2 or C lesions, 45.2 % bifurcations), did reveal similar early outcomes of BVS to second-generation DES and no evidence for increased scaffold thrombosis rates [24].

Other registries, mainly including less complex lesions, have provided good data on BVS safety and performance in true clinical experience. The German multicentre ASSURE registry showed low rates of cardiovascular death, myocardial infarction and target lesion revascularisation (0.5, 1.7 and 2.8 %, respectively) at 12 months after implantation (*n* = 183). No cases of scaffold thrombosis were observed [25]. Lastly, the ongoing multicentre ABSORB FIRST study was designed to enrol a high number of moderately complex ‘real-world’ patients. An interim analysis of the results from the first 800 patients at 30 days of follow-up demonstrated excellent device success rates (98.9 %), no cases of cardiovascular death and a low risk of definite or probable scaffold thrombosis (0.3 %) [26].

## BVS in ACS and STEMI patients: what do we know?

Immediately after clinical availability several institutions started treatment of more complex lesions with strict follow-up in several registries. We excluded STEMI patients as a large amount of thrombus is usually present which might result in malapposition if resolved in time. In the first registries a high number of NSTEMI patients where included of which a significant number of early angiographies demonstrated full vessel occlusion, an observation made by others [27]. After thrombus aspiration BVS implantation, performed in a similar fashion as in non-ACS patients and optical coherence tomography controlled, showed excellent apposition. This opened the door for BVS in STEMI patients.

In 2013, Wiebe and co-workers presented a first report on the short-term outcome of STEMI patients treated with an everolimus-eluting bioresorbable scaffold (predilatation). Twenty-five patients with 31 lesions were included with a procedural success rate of 97 % and major adverse cardiac event rate of 8.3 % during a mean follow-up period of 137 days [28].

Recently, our group reported the 30-day clinical outcome of the BVS STEMI First Study. In this prospective, single-arm, monocentre safety and feasibility study 49 STEMI patients were treated with a BVS (direct stenting in 32.7 % and predilatation in 67.3 %). The procedural success rate of BVS implantation was 97.9 %. TIMI flow III was obtained in 91.7 % of patients after BVS implantation. At 30 days, the major adverse cardiac event rate was 2.6 % (one patient with a non-Q-wave myocardial infarction in a non-target vessel). Target lesion failure (composite of cardiac death, target-vessel myocardial infarction or ischaemia-driven target lesion revascularisation) did not occur and there were no cases of scaffold thrombosis [29].

Additionally, in the prospective Prague 19 trial, BVS were implanted in consecutive STEMI patients from December 2012 until August 2013. The authors recently reported on 41 patients who received a BVS compared with a control group who were implanted with a drug-eluting or bare metal stent (*n* = 57) [30]. The BVS device success rate was 98 %. There were two events in the BVS group: one early (day 13) scaffold thrombosis after stopping aspirin and ticagrelor for which the patient underwent re-PCI and one non-target vessel myocardial infarction after a staged procedure with a DES. Four events (one cardiac death, two patients with unstable angina due to stent thrombosis and one myocardial infarction in a non-target vessel) were witnessed in the control group (95 % for BVS and 93 % for the control group, *p* = 0.674).

Recently, an update of the Prague-19 study, comprising 76 STEMI patients implanted with BVS, was presented during EuroPCR 2014, showing a target lesion revascularisation of 1.3 %, and a stent thrombosis and major adverse cardiac event rate of 1.3 and 2.6 %, respectively, with an average follow-up of about 6 months [30].

Concerning acute coronary syndromes, Gori et al. reported the short-term results in 150 consecutive patients (unstable angina 16 %, NSTEMI 40 %, STEMI 44 %), treated with in total 194 BVS between May 2012 and July 2013. These patients were compared with 103 consecutive control patients who received a DES (XIENCE Prime). Major adverse cardiac event rates at 30 days and 6 months were similar between the two groups. Scaffold thrombosis occurred in three BVS patients and two DES patients within the first month [31].

Also the POLAR ACS study (100 patients; unstable angina 46 %, NSTEMI 38 %, STEMI 16 %), reported an excellent device success rate (100 %) with limited (3.0 %) in-hospital major adverse cardiac event rate (due to two peri-procedural myocardial infarctions and one non-target vessel revascularisation). After one year, there was one additional myocardial infarction due to a definite scaffold thrombosis after discontinuatation of DAPT and another case of target lesion revascularization [32].

Overall the first trials, although still on a small number of patients, suggest that implantation of BVS in STEMI patients is feasible and safe, with early outcomes comparable to drug-eluting metal stents. However, these preliminary data need to be confirmed in future larger randomised controlled trials.

## General conclusion

Bioresorbable coronary artery scaffolds are the next step in PCI introducing the concept of the natural healing following PCI without leaving foreign body material in situ. The first-generation devices have shown encouraging results in selected patient studies up to the point of full bioresorption, supporting the introduction in regular patient care. During their introduction in daily clinical practice outside the previously selected patient groups a careful approach should be followed where outcome is continuously monitored.

### Conflict of interest

The institution Erasmus MC received research grants from Abbott. Robert-Jan van Geuns and Nicolas van Mieghem have received speakers fees from Abbott. Yoshinobu Onuma received a minor lecture fee from Abbott Vascular and is a member of the Advisory Board of Abbott Vascular. All other authors have no conflicts of interest to declare.
